# Supplementary material for: Predicting a failure of postoperative thromboprophylaxis in non-small cell lung cancer: A stacking machine learning approach
Source: PLoS One. 2025 Apr 1;20(4):e0320674. doi: 10.1371/journal.pone.0320674 (PMC11960935; doi:10.1371/journal.pone.0320674)
Supplement: Attachment — Submitted filename: responce letter2.docx [file pone.0320674.s003.docx]

Dear Editor，

We are truly grateful to your critical comments and thoughtful suggestions on our manuscript (Predicting A Failure of Postoperative Thromboprophylaxis in Non-Small Cell Lung Cancer: A Stacking Machine Learning Approach). Based on these comments and suggestions, we have made careful modifications on the original manuscript. We hope the new manuscript will meet your magazine’s standard. You will find our point-by-point responses to the comments/ questions as following.

Journal Requirements:

1. We notice that your manuscript file was uploaded on March 2024. Please can you upload the latest version of your revised manuscript as the main article file, ensuring that does not contain any tracked changes or highlighting. This will be used in the production process if your manuscript is accepted. Please follow this link for more information: http://blogs.PLOS.org/everyone/2011/05/10/how-to-submit-your-revised-manuscript/

Thanks for the good advices from editors. We upload the latest version of our revised manuscript as the main article file, ensuring that does not contain any tracked changes or highlighting.

2. Please note that funding information should not appear in the Acknowledgments section/Funding section or Any other areas of your Manuscript. We will only publish funding information present in the Funding Statement section of the online submission form. Please remove any funding-related text from the manuscript.

Thanks for the good advices from editors. We remove it from our manuscript.

3. Thank you for stating the following financial disclosure:

This work was supported by the Key Development Plan of XingTai (ZC20301 to Junjie Zhang and 2022ZC271 to Ligang Hao).

Please state what role the funders took in the study. If the funders had no role, please state: ""The funders had no role in study design, data collection and analysis, decision to publish, or preparation of the manuscript.""

If this statement is not correct you must amend it as needed.

Please include this amended Role of Funder statement in your cover letter; we will change the online submission form on your behalf.

Thanks for the good advices from editors. We upload the latest version of our cover letter which include the role of the funders took in the study.

4. Please amend the title either on the online submission form or in your so that they are identical.

Thanks for the good advices from editors. We recheck all titles and amend the title which was not identical.

Thanks for the good advices from editors, we submitted it as a supplemental figure.

Thanks for addressing the comments!

We hope that these revisions are satisfactory and that the revised version will be acceptable for publication in PLOS ONE.

Thank you very much for your work concerning our paper.

Wish you all the best!

Sincerely yours
